# Increased Concentrations of Circulating Soluble MHC Class I-Related Chain A (sMICA) and sMICB and Modulation of Plasma Membrane MICA Expression: Potential Mechanisms and Correlation With Natural Killer Cell Activity in Systemic Lupus Erythematosus

**DOI:** 10.3389/fimmu.2021.633658

**Published:** 2021-05-03

**Authors:** Baptiste Hervier, Matthieu Ribon, Nadine Tarantino, Julie Mussard, Magali Breckler, Vincent Vieillard, Zahir Amoura, Alexander Steinle, Reinhild Klein, Ina Kötter, Patrice Decker

**Affiliations:** ^1^ INSERM U1135, CNRS ERL 8255, Centre d’Immunologie et des Maladies Infectieuses (CIMI-Paris), Sorbonne Université, Paris, France; ^2^ Service de Médecine Interne-Maladies Systémiques, Hôpital Saint-Louis, Assistance Publique Hôpitaux de Paris, Paris, France; ^3^ Li2P, University Sorbonne Paris Nord, Bobigny, France; ^4^ INSERM UMR 1125, Bobigny, France; ^5^ Hôpital de la Pitié-Salpêtrière, Service de Médecine Interne 2, Centre National de Référence Maladies Systémiques Rares, Lupus et Syndrome des Anticorps Antiphospholipides, Centre National de Référence Histiocytoses, Sorbonne Université, Assistance Publique Hôpitaux de Paris, Paris, France; ^6^ Institute for Molecular Medicine, Goethe-University Frankfurt am Main, Frankfurt am Main, Germany; ^7^ Frankfurt Cancer Institute, Frankfurt am Main, Germany; ^8^ Department of Hematology, Oncology, Clinical Immunology and Rheumatology, University Hospital Tübingen, Tübingen, Germany; ^9^ Division of Rheumatology and Systemic Inflammatory Diseases, University Hospital Hamburg-Eppendorf (UKE), Hamburg, Germany

**Keywords:** soluble MICA, plasma membrane MICA, natural killer cells, monocytes, systemic lupus erythematosus, extracellular chromatin

## Abstract

Systemic lupus erythematosus (SLE) is a severe autoimmune disease of unknown etiology. The major histocompatibility complex (MHC) class I-related chain A (MICA) and B (MICB) are stress-inducible cell surface molecules. MICA and MICB label malfunctioning cells for their recognition by cytotoxic lymphocytes such as natural killer (NK) cells. Alterations in this recognition have been found in SLE. MICA/MICB can be shed from the cell surface, subsequently acting either as a soluble decoy receptor (sMICA/sMICB) or in CD4^+^ T-cell expansion. Conversely, NK cells are frequently defective in SLE and lower NK cell numbers have been reported in patients with active SLE. However, these cells are also thought to exert regulatory functions and to prevent autoimmunity. We therefore investigated whether, and how, plasma membrane and soluble MICA/B are modulated in SLE and whether they influence NK cell activity, in order to better understand how MICA/B may participate in disease development. We report significantly elevated concentrations of circulating sMICA/B in SLE patients compared with healthy individuals or a control patient group. In SLE patients, sMICA concentrations were significantly higher in patients positive for anti-SSB and anti-RNP autoantibodies. In order to study the mechanism and the potential source of sMICA, we analyzed circulating sMICA concentration in Behcet patients before and after interferon (IFN)-α therapy: no modulation was observed, suggesting that IFN-α is not intrinsically crucial for sMICA release *in vivo*. We also show that monocytes and neutrophils stimulated *in vitro* with cytokines or extracellular chromatin up-regulate plasma membrane MICA expression, without releasing sMICA. Importantly, in peripheral blood mononuclear cells from healthy individuals stimulated *in vitro* by cell-free chromatin, NK cells up-regulate CD69 and CD107 in a monocyte-dependent manner and at least partly *via* MICA-NKG2D interaction, whereas NK cells were exhausted in SLE patients. In conclusion, sMICA concentrations are elevated in SLE patients, whereas plasma membrane MICA is up-regulated in response to some lupus stimuli and triggers NK cell activation. Those results suggest the requirement for a tight control *in vivo* and highlight the complex role of the MICA/sMICA system in SLE.

## Introduction

Systemic lupus erythematosus (SLE) is a rheumatic, inflammatory and autoimmune disease of unknown etiology targeting mainly the skin, joints, kidneys and the central nervous system. It affects essentially women and is triggered by a combination of genetic and environment factors. SLE involves immune dysregulation and is characterized by the production of numerous autoantibodies. Among the target recognized, chromatin is a major lupus autoantigen. Indeed, IgG3 anti-nucleosome autoantibodies are associated with active disease (1) and nucleosome-specific autoreactive T-helper cells are found in SLE patients (2). Moreover, increased concentrations of circulating nucleosomes are detected in SLE patients (3) and are correlated with disease activity (4). In addition, extracellular nucleosomes behave like damage-associated molecular patterns (DAMP), activating key cells involved in lupus pathogenesis like dendritic cells (5) and polymorphonuclear neutrophils (PMN) (6), leading to interferon (IFN)-α secretion (7) and the release of soluble CEACAM8 (8). Moreover, nucleosomes are detected in glomerular deposits in human lupus nephritis (9) and bind laminin β1, which is aberrantly expressed in the glomerular basement membrane during lupus nephritis (10).

Natural killer (NK) cells are involved in killing target cells but they produce also large numbers of cytokines that shape immune responses. Several of those cytokines have immunoregulatory functions. Especially, by producing different cytokines, including interleukin (IL)-10, IFN-γ and tumor necrosis factor (TNF), NK cells may also have important immunoregulatory effects. NK cells may also behave as antigen-presenting cells and regulate T-cell activation (11). Whether NK cells contribute to SLE development is still unclear. Proportions, absolute numbers and cytotoxicity of circulating NK cells are decreased in SLE patients, particularly in patients with active disease, e.g. lupus nephritis, and especially CD226^+^ NK cells (12–16). On the contrary, CD226^+^ NK cells infiltrate the kidneys of predisease lupus mice and these infiltrating NK cells display an activated phenotype and a marked ability to produce cytokines and cytotoxic granules, suggesting they may mediate tissue injury (16). Likewise, NK cells exhibit a phenotype of activated cells in patients with active SLE and have a higher capacity to produce IFN-γ (17). NK cells also participate in SLE pathogenicity by enhancing IFN-α production by plasmacytoid dendritic cells in response to immune complexes, although lupus NK cells display a lower capacity (18).

NKG2D is an activating receptor expressed on most NK cells, CD8^+^ T cells, γδ T cells but not on normal CD4^+^ T cells. Ligand engagement by NKG2D activates NK cells and costimulates effector T cells (19, 20). NKG2D expression is controlled by ligand-induced down-regulation (21). Among NKG2D ligands, are the stress-inducible major histocompatibility complex (MHC) class I-related chain A (MICA) and B (MICB) and ULBP proteins (19, 22, 23). MIC can also be shed from the cell surface to generate soluble MIC (sMIC) (21, 24). However, according to the context and the cells analyzed, sMIC can behave either as a decoy or an activating molecule.

Abnormal NKG2D ligand expression has been implicated in the initiation or maintenance of several autoimmune and/or inflammatory diseases, including SLE and rheumatoid arthritis (RA). In RA, MIC-expressing synoviocytes may expand autoreactive CD4^+^CD28^-^ T cells, which express NKG2D (whereas it is absent on normal CD4^+^ T cells), promoting inflammation (25). In SLE, NKG2D-mediated immune responses may be immune-modulatory rather than tissue-damaging. Increased frequencies of NKG2D^+^CD4^+^ T cells, producing IL-10, are observed in juvenile-onset SLE and inversely correlate with disease activity (26). Increased sMICB concentrations have been reported in the plasma of those patients; they correlate positively with the percentage of NKG2D^+^CD4^+^ T cells, but negatively with disease activity, suggesting that sMICB is involved in the expansion of this immunosuppressive subset (27). However, NK cells have not been investigated in these studies, which in addition were not dedicated to adult SLE.

Thus, few studies have investigated the role MICA/B in SLE pathogenesis, and even less focused on the soluble forms. Moreover, some data are contradictory. Likewise, the consequences on NK cells are poorly documented. Altogether, those data show that the potential contribution of NK cells to SLE is complex and requires further investigations. For example, it has been suggested that NK cells may also link innate and adaptive immunity, e.g. by interacting with dendritic cells. Therefore, we have analyzed the role of both MICA and MICB, either as plasma membrane or soluble forms, their modulation and the consequences on NK cell phenotype and activity, focusing on SLE.

## Methods

### Human Samples

Blood was collected from healthy volunteers, SLE patients [fulfilling four or more of the revised American College of Rheumatology (ACR) classification criteria (28)], RA patients (fulfilling the American College of Rheumatology-European League Against Arthritis (EULAR) criteria, analyzed at baseline and 3-6 months after B-cell depletion therapy by rituximab), primary Sjögren’s syndrome (pSS) patients (fulfilling ACR/EULAR classification criteria), Behcet patients (fulfilling ISG (International Study Group) for Behcet’s disease criteria, analyzed at baseline and 3-10 weeks after IFN-α therapy) and leukemia patients (5 patients with acute myeloid leukemia, 2 patients with acute lymphoid leukemia and 3 patients with chronic lymphoid leukemia). A control group was composed of patients not treated by immunosuppressive drugs and suffering from fibromyalgia (n = 8), uveitis (n = 5), Raynaud’s syndrome (n = 1), chronic fatigue syndrome (n = 1), tinnitus (n = 1) or inner ear hearing impairment (n = 2). Blood samples were used to prepare plasma or serum or to isolate leukocytes. For *in vitro* studies with purified nucleosomes, cells were isolated from untreated (exclusion criterion) inactive SLE patients (SLEDAI score ≤4). Informed consents were collected. Human experiments were approved by the local ethics committees [CPP Paris Ile de France (NI-2016-11-01, Bobigny), CPP IDF VI (June 26^th^, 2012, Paris), references 146/2001V, 386/2006V and 647/2016B02 (Tübingen)]. Alternatively, blood samples from healthy volunteers were obtained from the Etablissement Français du Sang (EFS, contract 18/EFS/033).

### Chromatin Purification

Mono-nucleosomes were purified from calf thymus under sterile conditions as previously described (5, 6, 29). Briefly, nuclei were isolated using a Dounce tissue homogenizer and then digested with micrococcal nuclease (Sigma-Aldrich). The reaction was stopped by EDTA and nuclei were lysed. After centrifugation, the supernatant (which contains chromatin fragments of different sizes) was collected and further purified by ultracentrifugation on 5-29% sucrose gradients. As a control, an empty sucrose gradient was loaded with the lysis buffer only. After centrifugation, several fractions were collected for both the chromatin-loaded and the buffer-loaded gradients. The latter was used as a negative control in cell culture (and is defined as the nucleosome purification buffer). All chromatin fractions were analyzed by agarose gel electrophoresis (1.5%) and SDS-PAGE (18%). Fractions containing mono-nucleosomes (180 base pairs of DNA and the five histones H1, H2A, H2B, H3 and H4) were harvested and used in the present study. It should be noted that free self DNA is not strongly immunogenic and that core histones are 100% conserved between human and calf.

### Cell Isolation and Culture

PMN and peripheral blood mononuclear cells (PBMC) were freshly isolated by dextran sedimentation (Axis Shield) from peripheral blood as previously described (6, 8). Contaminating red blood cells were lysed using cold ACK (NH_4_Cl, KHCO_3_, EDTA) hypotonic buffer. Alternatively, PBMC were prepared by Ficoll-Hypaque density centrifugation. Monocytes were either purified from PBMC by negative selection using magnetic sorting (Miltenyi Biotec) or were further enriched by a second density centrifugation through a 46% Percoll cushion (30). NK cells were isolated from PBMC by negative selection by magnetic sorting (Miltenyi Biotec). In some experiments, PBMC were depleted from monocytes using CD14 magnetic beads (Miltenyi Biotec). In all cases, cell purity was estimated by flow cytometry.

To estimate plasma membrane MICA/B up-regulation and soluble MICA/B release, PBMC, PMN or monocytes were cultured with 5-40 µg/ml purified mono-nucleosomes or the purification buffer (empty gradient), 5-45 ng/ml granulocyte-macrophage colony-stimulating factor (GM-CSF, ImmunoTools), 5-40 ng/ml granulocyte colony-stimulating factor (G-CSF, ImmunoTools), 20-60 ng/ml IL-17A (ImmunoTools) or lipopolysaccharides (LPS, from *Salmonella typhimurium*, a Toll-like receptor (TLR) 4 agonist, 5 ng/ml (PMN) or 0.5-1 µg/ml (monocytes and PBMC), Sigma-Aldrich) for 15-20 hours. Cell culture supernatants were analyzed by ELISA and cells by flow cytometry.

To determine if NK cells are directly activated by nucleosomes, NK cells purified by negative magnetic sorting were cultured with 2.5-100 µg/ml purified mono-nucleosomes or phorbol myristate acetate (PMA) and ionomycin for 24 hours and activation was estimated by flow cytometry and ELISA.

To estimate NK cell activation in PBMC or monocyte-depleted PBMC, freshly isolated PBMC (from healthy individuals or from hydroxychloroquine-free SLE patients) were incubated overnight with or without purified mono-nucleosomes (25 µg/ml) or a TLR9 agonist (a synthetic oligonucleotide (ODN) containing unmethylated CpG motifs, CpG-ODN-A 2216, InvivoGen, 5µM). For two healthy donors, PBMC were depleted from monocytes, using Miltenyi CD14 microbeads, and the absence of monocytes was finally attested by flow cytometry, showing a percentage of CD14^+^ cells < 1%. NK cell activation was estimated after 14 hours by flow cytometry. In addition, 5 hour-degranulation assays were performed as previously described: 5x10^5^ PBMC or PBMC depleted from monocytes (stimulated as described above) were incubated with human leukocyte antigen (HLA) class I-negative K562 target cells (ATCC CCL243) at 1:1 ratio, in the presence of anti-CD107a monoclonal antibody (mAb) to monitor degranulation. After one hour of incubation, monensin (GolgiStop, BD Biosciences) and brefeldin A (GolgiPlug, BD Biosciences) were added during the remaining four hours and intracellular cytokine production by NK cells was also estimated, after permeabilization.

C1R-MICA and C1R-mock transfectants were previously described (24).

### ELISA

Soluble MICA/B was measured as previously described (31) using recombinant sMICA*04 and sMICB*02 as standards, except that the peroxidase-conjugated anti-mouse IgG2a antibody was diluted 1:10000. Soluble MICA/B concentrations in serum/plasma were measured in triplicate for each individual donor. Then, the mean of the triplicate of each sample was used in figures and for statistical analyses. Concentrations in different donor groups are depicted as mean ± SEM (figures) and mean ± SD (tables) of all donors. For cell culture supernatants, the mean ± SD of data pooled from five independent experiments is shown.

IFN-γ secretion by pure NK cells and IL-6 secretion by monocytes were quantified by sandwich ELISA using mAb pair and streptavidin-peroxidase conjugate (BD Biosciences) and according to the manufacturer’s instructions. Concentrations in cell culture supernatants are depicted as the mean of duplicates from one representative experiment (NK cells) or as mean ± SD of data pooled from five independent experiments.

### Flow Cytometry

Plasma membrane MICA expression was estimated by staining with anti-MICA AMO1 mAb or mouse IgG1 isotype control [10 µg/ml (24)] followed by a PE-conjugated goat anti-mouse IgG secondary antibody (Jackson ImmunoResearch). Monocytes, lymphocytes and PMN were gated according to FSC/SSC parameters and staining with mAb specific for CD14 (clone M5E2), CD3/CD19 (clones UCHT1 and HIB19) and CD66b (clone G10F5), respectively (all FITC-conjugated, BD Biosciences). Dead cells were excluded using 7-amino-actinomycin D (7-AAD, BD Biosciences).

Activation of NK cells (defined as CD3^-^CD56^+^) in PBMC was evaluated through the positivity of CD69 or CD69 expression levels (normalized to non-stimulated cells) as well as cell surface expression of NKG2D in the different cell culture conditions after staining with eFluor 780-conjugated anti-CD3 (clone UCHT1, eBioscience), PE-Vio 770-conjugated anti-CD56 (clone REA196, Miltenyi Biotec), ECD-conjugated anti-CD69 (clone TP1.55.3, Beckman Coulter) and APC-conjugated anti-NKG2D (CD314, clone BAT221, Miltenyi Biotec) mAb. Dead cells were excluded using the fixable viability dye eFluor 506 (Invitrogen). CD3^-^CD56^+^ NK cells were also analyzed in functional assays. To estimate degranulation and cytokine production, cells were then stained with FITC-conjugated anti-CD107a mAb (clone H4A3, BD Biosciences) and thereafter were fixed/permeabilized with cytofix/cytoperm kit (BD Biosciences) and then intracellularly stained with Alexa Fluor 700-conjugated anti-IFN-γ mAb (clone B27, BD Biosciences).

CD69 expression by purified NK cells was analyzed after staining with a PE-conjugated anti-CD69 mAb (clone FN50) or a PE-conjugated mouse IgG1 isotype control (both from BD Biosciences).

Cell purity and monocyte depletion were estimated by flow cytometry after staining for CD56^+^CD3^-^ (NK cells), CD14^+^ (monocytes) and CD66b^+^ (PMN) cells.

All stainings were performed at 4°C and according to classical protocols. Cells were analyzed on a four-color FACSCalibur (Becton Dickinson) or a nine-color Gallios (Beckman Coulter) apparatus. Data were evaluated with CellQuest Pro or FlowJo software (BD Biosciences). Results are presented as mean fluorescence intensity (MFI) or percentages of cells positive for the indicated markers among all living cells. Data are depicted as representative experiments or as mean ± SEM in pooled data.

### Statistical Analysis

In all figures, the legend indicates whether representative experiments and/or pooled data are shown, the number of independent experiments, the number of samples tested and the number of independent chromatin preparations.

Soluble MICA concentrations in the plasma of healthy individuals and SLE patients were compared using a two-tailed Mann Whitney test. Soluble MICA/B concentrations in the sera of healthy individuals, controls, and patients with SLE, pSS, RA, leukemia or Behcet’s disease were compared using a Kruskal-Wallis test with Dunn’s multiple comparison test. Concentrations of circulating sMICA in untreated versus treated SLE patients were compared using a two-tailed Mann Whitney test. Differences in sMICA concentrations between SLE patients positive or negative for antibodies of interest were tested using a two-tailed Mann Whitney test. Differences in sMICA concentrations in patients before/after rituximab or IFN-α therapy were tested using a two-tailed Wilcoxon matched-pairs signed rank test. Soluble MICA concentration in sera versus plasmas were compared using a two-tailed unpaired *t*-test after having checked that both groups follow a Gaussian distribution and have similar variances. Soluble MICA concentrations in culture supernatants from monocytes and C1R-MICA transfectants were compared using a two-tailed unpaired *t*-test with Welch’s correction (after having checked that both groups follow a Gaussian distribution but don’t have similar variances). The correlation between sMICA concentrations and percentages of monocytes was assessed using a two-tailed Spearman correlation test. Soluble MICA concentrations measured in the same samples at two different time points were compared using a two-tailed paired *t*-test after having checked that both groups follow a Gaussian distribution and the correlation was tested using a two-tailed Pearson correlation test.

Additionally, when comparing two groups of donors for circulating sMICA concentrations, receiver-operator characteristic (ROC) curves were established. The area under the curve (AUC) and the p value are depicted for each ROC curve.

Percentages of NK cells positive for CD69, CD107a or IFN-γ among PBMC in response to nucleosomes or the TLR9 agonist versus unstimulated cells were compared using a two-tailed paired *t*-test (healthy individuals, after having checked that both groups follow a Gaussian distribution) or a two-tailed Wilcoxon matched-pairs signed rank test (SLE patients). CD69 expression levels in response to nucleosomes were normalized to non-stimulated cells and analyzed using a one sample *t*-test against the expected value of 1 (in healthy individuals or SLE patients). Comparison of CD69 expression levels between healthy individuals and SLE patients was performed using a two-tailed unpaired *t*-test.

Data were analyzed using GraphPad Prism software (p ≤ 0.05 was considered significant).

## Results

### Concentrations of Circulating Soluble MICA and Soluble MICB Are Increased in SLE Patients as Well as in Patients Suffering From Primary Sjögren’s Syndrome

The role of NK cells in SLE and the mechanisms involved are still not resolved. Very few studies analyzed the influence of the activating receptor NKG2D and its ligands, either as plasma membrane or soluble forms. To better understand NK cell activity in SLE, we investigated the roles of plasma membrane and soluble sMICA/B, their modulation and the consequences on NK cells.

**Figure 1:**
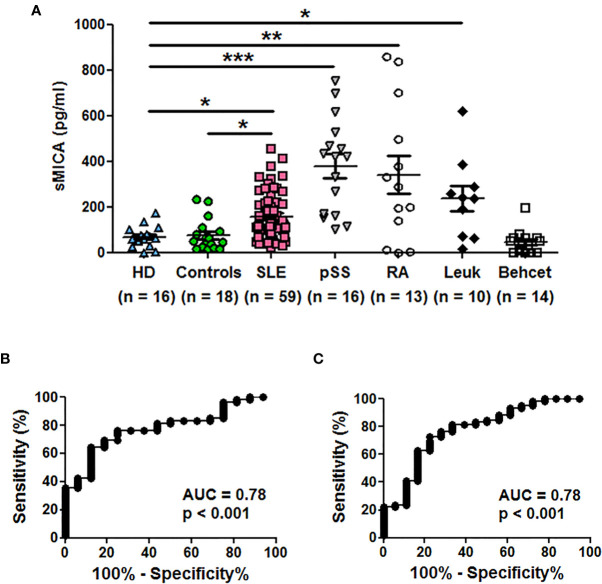
Soluble MICA concentrations are increased in sera of SLE patients and primary Sjögren’s syndrome patients in comparison to healthy individuals or control patients. **(A)** Serum concentrations of sMICA have been determined by ELISA. Each dot represents an independent donor. HD, healthy donors; Controls, patients not treated by immunosuppressive drugs and suffering from fibromyalgia, uveitis, Raynaud’s syndrome, chronic fatigue syndrome, tinnitus or inner ear hearing impairment; SLE, systemic lupus erythematosus; pSS, primary Sjögren’s syndrome; RA, rheumatoid arthritis; Leuk, leukemia. Mean and SEM of all donors are shown. *p < 0.05; **p < 0.01; ***p < 0.001 (Kruskal-Wallis test with Dunn’s multiple comparison test). Data were also significant when compared to the control patient group but significance is not depicted for clarity. **(B)** ROC curve illustrating the power of sMICA for discrimination between SLE patients and healthy individuals. AUC, area under the curve. **(C)** ROC curve illustrating the power of sMICA for discrimination between SLE patients and the control patient group.

We first measured sMICA concentrations in a series of sera from SLE patients (n = 59) in comparison to healthy individuals (n = 16). Soluble MICA was detected in both donor groups but sMICA concentrations were significantly higher in SLE patients than in healthy individuals [**Table 1** and **Figure 1A**, p <0.05 (Kruskal-Wallis test with Dunn’s multiple comparison test)]. The significant difference in sMICA concentrations between SLE patients and healthy individuals is illustrated by the ROC curve (**Figure 1B**, AUC = 0.78, p <0.001). SLE patients were also compared to a control group composed of 18 patients not treated by immunosuppressive drugs and suffering from fibromyalgia, uveitis, Raynaud’s syndrome, chronic fatigue syndrome, tinnitus or inner ear hearing impairment. Serum sMICA concentrations were significantly higher in SLE patients than in control patients [**Table 1** and **Figure 1A**, p <0.05 (Kruskal-Wallis test with Dunn’s multiple comparison test)] and the ROC curve illustrates the significant difference (**Figure 1C**, AUC = 0.78, p <0.001). It should be noted that sMICA concentrations were not different between healthy individuals and the control patient group. Serum sMICA concentrations were next compared to those measured in patients with pSS (n = 16) or Behcet’s disease (n = 14) as well as patients previously reported with elevated sMICA concentrations, e.g. patients with leukemia (n = 10) or RA (n = 13). Interestingly, serum sMICA concentrations were significantly increased in pSS (p <0.001), as well as in RA (p < 0.01) and leukemia patients (p <0.05) as expected, in comparison to healthy individuals, with pSS patients showing the highest mean concentration (**Figure 1A** and **Table 1**, Kruskal-Wallis test with Dunn’s multiple comparison test), whereas no significant difference was observed with Behcet patients. As with SLE, serum sMICA concentrations were also significantly higher in patients with pSS (p <0.001), RA (p <0.01) and leukemia (p <0.05) in comparison to the control patient group (Kruskal-Wallis test with Dunn’s multiple comparison test, **Table 1**).

**Table 1:** Concentrations of soluble MICA in the serum of healthy individuals and patients with diverse pathologies.

	HD (n = 16)	Controls (n = 18)	SLE (n = 59)	pSS (n = 16)	RA (n = 13)	Leukemia (n = 10)	Behcet (n= 14)
**sMICA (pg/ml)**	68.66 ± 45.29	74.92 ± 67.38	157.8 ± 104.7	380.0 ± 207.3	341.3 ± 302.2	237.0 ± 176.6	45.19 ± 51.59
p vs HD		n.s.	<0.05	<0.001	<0.01	<0.05	n.s.
p vs controls	n.s.		<0.05	<0.001	<0.01	<0.05	n.s.

Concentrations of sMICA have been determined by ELISA. The mean and SD are indicated. Groups were compared using a Kruskal-Wallis test with Dunn’s multiple comparison test. HD, healthy individuals; Controls, patients not treated by immunosuppressive drugs and suffering from fibromyalgia, uveitis, Raynaud’s syndrome, chronic fatigue syndrome, tinnitus or inner ear hearing impairment; SLE, systemic lupus erythematosus; pSS, primary Sjögren’s syndrome; RA, rheumatoid arthritis; n.s., not significant.

**Figure 2:**
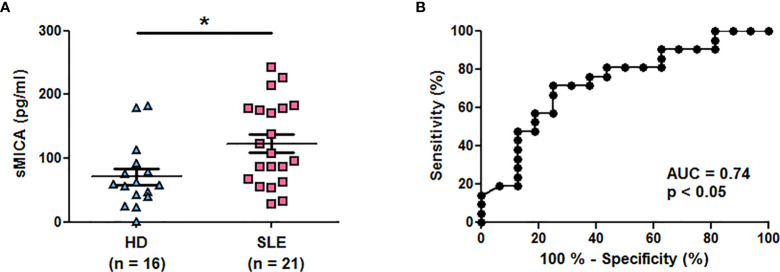
Higher plasma concentrations of soluble MICA in SLE patients in comparison to healthy individuals. **(A)** Plasma concentrations of sMICA have been determined by ELISA. Each dot represents an independent donor. HD, healthy donors; SLE, systemic lupus erythematosus. Mean and SEM of all donors are shown. *p < 0.05 (two-tailed Mann Whitney test). **(B)** ROC curve illustrating the power of sMICA for discrimination between SLE patients and healthy individuals. AUC, area under the curve.

Results were confirmed in a second series of healthy individuals (n = 16) and SLE patients (n = 21) and we tested plasma instead of sera. Plasma sMICA were also significantly increased in SLE patients in comparison to healthy individuals (**Figure 2A**, mean ± SD = 123.1 ± 65.69 versus 71.24 ± 50.69 pg/ml, respectively; p < 0.05 (two-tailed Mann Whitney test)). The ROC curve illustrates the significant difference (**Figure 2B**, AUC = 0.74, p < 0.05). Moreover, reproducibility was demonstrated by testing at two different time points part of the serum samples (n = 18) from SLE patients. Indeed, similar concentrations were measured in both tests (**Supplementary Figure 1**, mean ± SD = 215.1 ± 123.0 versus 220.0 ± 156.9 pg/ml, p = 0.77, two-tailed paired *t*-test) and were correlated (r^2^ = 0.81; p <0.0001, two-tailed Pearson correlation test).

**Figure 3:**
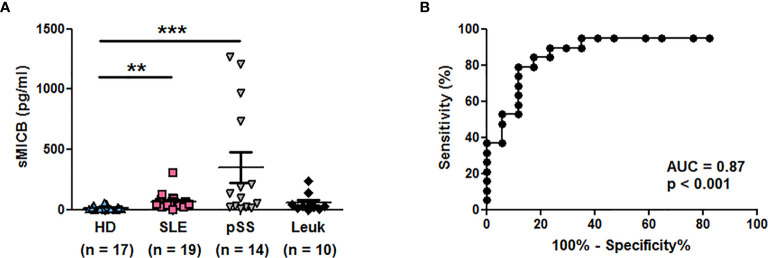
Serum concentrations of soluble MICB in different patient groups. **(A)** Concentrations of sMICB have been determined by ELISA in the serum of healthy donors (HD), lupus patients (SLE), patients with primary Sjögren’s syndrome (pSS) and patients with leukemia (Leuk). Each dot represents an independent donor. Mean and SEM of all donors are shown. **p < 0.01; ***p < 0.001 (Kruskal-Wallis test with Dunn’s multiple comparison test). **(B)** ROC curve illustrating the power of sMICB for discrimination between SLE patients and healthy individuals. AUC, area under the curve.

We measured in addition concentrations of sMICB in patient groups of interest. As observed with sMICA, significantly elevated sMICB concentrations were observed in sera of SLE (n = 19, p < 0.01) and especially pSS patients (n = 14, p < 0.001) in comparison to healthy individuals (n = 17, Kruskal-Wallis test with Dunn’s multiple comparison test, **Figure 3A** and **Table 2**). The ROC curve showing the significant difference between SLE patients and healthy individuals is depicted in **Figure 3B** (AUC = 0.87, p <0.001).

**Table 2:** Comparison of sMICB concentrations in the serum of healthy individuals and different patient groups.

	HD (n = 17)	SLE (n = 19)	pSS (n = 14)	Leukemia (n = 10)
**sMICB (pg/ml)**	15.06 ± 18.02	67.39 ± 64.53	350.20 ± 472.0	56.38 ± 75.95
p		<0.01	<0.001	n.s.

Concentrations of sMICB have been determined by ELISA in healthy individuals (HD) and in patients with SLE, systemic lupus erythematosus; pSS, primary Sjögren’s syndrome; or leukemias. The mean and SD are indicated. Groups were compared using a Kruskal-Wallis test with Dunn’s multiple comparison test. P values versus healthy individuals are shown. n.s., not significant.

### Associations Between Circulating sMICA Concentrations and Clinical Parameters in SLE Patients

From here, we focused on sMICA in SLE. Clinical data were analyzed according to circulating sMICA concentrations determined in both sera and plasmas of independent patients, because there was no significant difference between serum (n = 16) and plasma (n = 16) sMICA concentrations (p = 0.88, two-tailed unpaired *t*-test). First, to exclude any influence of the therapy on sMICA concentrations, we compared circulating sMICA concentrations in untreated SLE patients (n = 12) versus SLE patients under any therapy (n = 59); no significant difference was observed (p = 0.41, two-tailed Mann Whitney test). Among SLE patients, circulating sMICA concentrations were higher in patients positive for anti-SSB (**Table 3**, p <0.01, two-tailed Mann Whitney test) or anti-RNP (**Table 4**, p <0.05, two-tailed Mann Whitney test) antibodies than SLE patients negative for these antibodies. No significant differences were observed between SLE patients positive or negative for anti-SSA or anti-double-stranded DNA antibodies. No correlation was observed between sMICA concentrations and disease activity, as estimated using the SLEDAI score. Likewise, sMICA concentrations were not associated with kidney, skin or joint manifestations. Importantly, we could not detect anti-MICA IgG autoantibodies by ELISA, indicating that increased levels of circulating sMICA do not lead to the induction of anti-MICA autoimmunity in SLE patients.

**Table 3:** Increased circulating sMICA concentrations in SLE patients positive for anti-SSB antibodies.

	Anti-SSB-negative (n = 74)	Anti-SSB-positive (n = 6)
**sMICA (pg/ml)**	140.5 ± 93.17	250.3 ± 90.01

Concentrations of sMICA have been determined by ELISA in the serum or plasma of 80 SLE patients and compared between patients negative or positive for anti-SSB antibodies. The mean and SD are indicated. Groups were compared using a two-tailed Mann Whitney test (p < 0.01).

**Table 4:** Increased circulating sMICA concentrations in SLE patients positive for anti-RNP antibodies.

	Anti-RNP-negative (n = 75)	Anti-RNP-positive (n = 5)
**sMICA (pg/ml)**	139.8 ± 89.27	281.9 ± 119.2

Concentrations of sMICA have been determined by ELISA in the serum or plasma of 80 SLE patients and compared between patients negative or positive for anti-RNP antibodies. The mean and SD are indicated. Groups were compared using a two-tailed Mann Whitney test (p < 0.05).

### Interferon-α Is Not Involved in Increased Levels of Circulating Soluble MICA *In Vivo*

**Figure 4:**
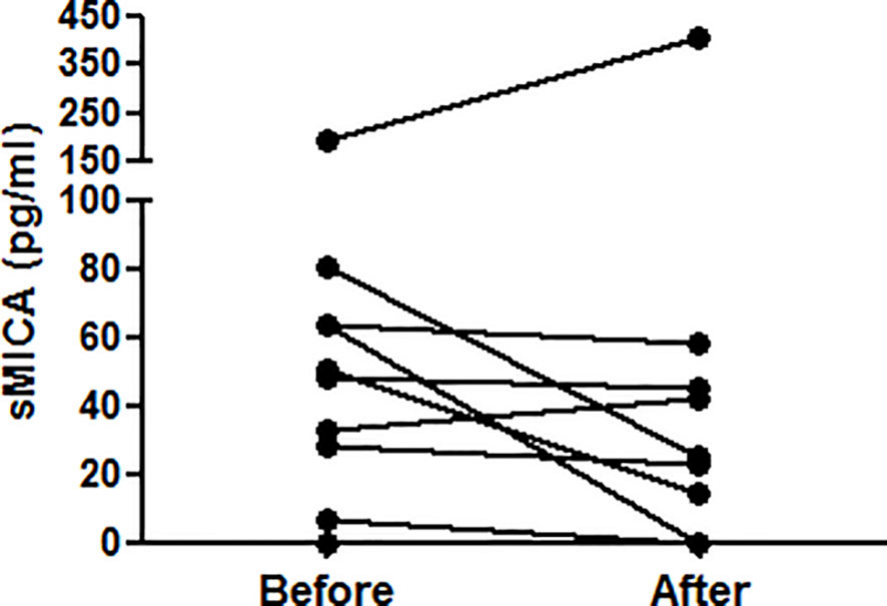
IFN-α does not induce elevated serum sMICA concentrations *in vivo*. Concentrations of sMICA in the serum of 14 Behcet patients have been determined by ELISA before and after IFN-α therapy. Each dot represents an independent patient. Serum sMICA was not detected in 4 patients (either before or after therapy). Serum sMICA concentrations were compared using a two-tailed Wilcoxon matched-pairs signed rank test.

After having compared different inflammatory and autoimmune conditions to SLE for circulating sMICA, we next investigated the potential mechanisms involved in increased concentrations of circulating sMICA *in vivo* and the major source of soluble MICA *in vivo*. We analyzed situations for which the therapy may allow approaching specific *in vivo* pathophysiological mechanisms. Because IFN-α is a key lupus cytokine, we investigated whether this cytokine may induce elevated serum sMICA concentrations *in vivo*. Indeed, IFN-α was reported to induce MICA expression, at least on dendritic cells (32). We therefore analyzed serum sMICA concentrations in 14 Behcet patients before and after IFN-α therapy (patients were treated for sight threatening panuveitis/retinal vasculitis, in accordance with EULAR recommendations). As shown above, very low sMICA concentrations were measured in the sera of Behcet patients (**Figure 1A** and **Table 1**, not significantly different from healthy individuals). Moreover, no statistically significant increase in serum sMICA concentrations was observed after IFN-α therapy (**Figure 4**). Only one patient displayed a clear increased sMICA concentration after therapy, whereas in four patients the concentration was decreased. Moreover, in four other patients, serum sMICA was not detected, either before or after therapy. In agreement with these results, we did not detect higher plasma sMICA concentrations in SLE patients tested positive for plasma IFN-α by ELISA in comparison to SLE patients with no detectable plasma IFN-α (data not shown). Similarly, because many different autoantibodies are produced in SLE and some of them are either a disease marker or even pathogenic, B lymphocytes play a key role in the disease. Using the same approach, we analyzed serum sMICA concentrations in a few patients also reported with elevated circulating sMICA concentrations, namely RA patients, before and after *in vivo* B-cell depletion therapy by rituximab (anti-CD20 antibody). Indeed, RA patients (who also produce pathogenic autoantibodies) are much more often treated by rituximab than SLE patients. However, no significant reduction of serum sMICA concentrations was observed after therapy (data not shown).

### Modulation of Plasma Membrane MICA Expression by Monocytes and Neutrophils and Consequences on Soluble MICA

To better understand the mechanism leading to high levels of circulating sMICA, we analyzed plasma membrane MICA expression, its modulation and whether increased expression leads to MICA shedding. We observed a positive and significant correlation between plasma sMICA concentrations and the percentage of blood monocytes *in vivo* in SLE patients (r = 0.52, p < 0.05, two-tailed Spearman correlation test). Moreover, we have previously reported that plasma membrane MICA expression, but not expression of MICB and ULPB molecules, is induced on human monocytes upon stimulation (30). Likewise, PMN are activated in SLE and, especially, they represent about 50% of blood leukocytes. Therefore, we focused on MICA expression and sMICA release by monocytes and PMN. Because we did not observe modulation of plasma membrane MICA expression by monocytes (30) as well as PMN (data not shown) in response to IFN-α, we investigated the effects of other cytokines and used TLR stimulation as a positive control for monocytes as previously reported (30). Moreover, we analyzed the impact of extracellular chromatin. Indeed, circulating chromatin, and especially mono-nucleosomes, represents a major autoantigen in SLE and, in addition, it acts like a DAMP. Particularly, nucleosomes trigger PMN activation (6, 7, 33).

**Figure 5:**
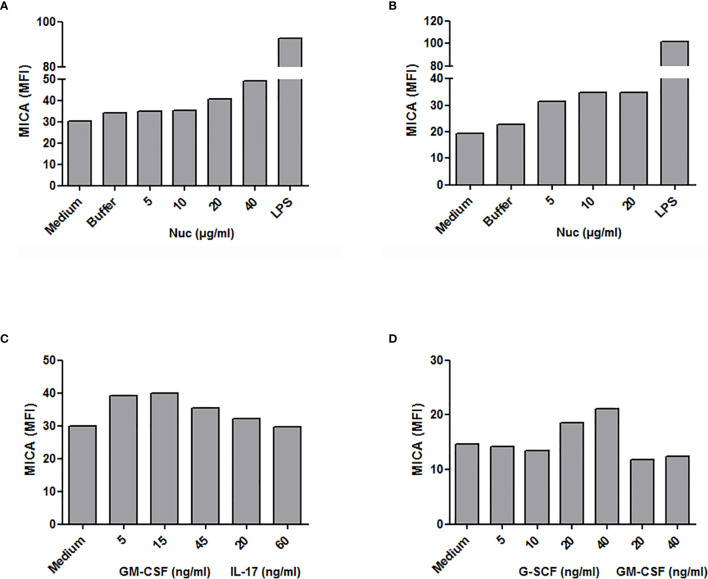
Modulation of plasma membrane MICA expression in monocytes and neutrophils. PBMC, monocytes or PMN were stimulated *in vitro* with purified mono-nucleosomes (Nuc), cytokines or lipopolysaccharides (LPS) and MICA expression was analyzed by flow cytometry. **(A)** PBMC were used and monocytes were gated. **(B, C)** Enriched monocytes. **(D)** Isolated PMN. In each panel, shown is one representative experiment of at least three independent experiments using cells from independent donors and independent nucleosome preparations. MFI, mean fluorescence intensity; Buffer, buffer used for nucleosome purification.

**Figure 6:**
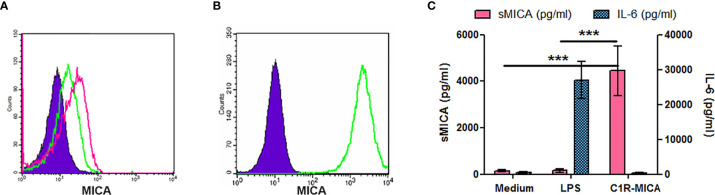
Only extreme plasma membrane MICA expression leads to sMICA release. Cells were cultured for 15 hours and MICA expression was analyzed by flow cytometry **(A, B)**, while sMICA release as well as IL-6 secretion were estimated by ELISA **(C)**. **(A)** MICA expression by enriched monocytes, either unstimulated (green histogram) or after LPS stimulation (pink histogram). As a negative control, monocytes were stained with an isotype control instead of the MICA-specific monoclonal antibody (filled purple histogram). **(B)** MICA expression by C1R-MICA transfectants. Green histogram, MICA staining; filled purple histogram, isotype control staining. **(C)** Soluble MICA release and IL-6 secretion by monocytes cultured in medium only or supplemented with 0.5 µg/ml LPS and by C1R-MICA transfectants. Shown is one representative experiment of at least five independent experiments using cells from independent donors **(A, B)** or data pooled from five independent experiments (C, mean and SD are depicted). ***p < 0.0001 (two-tailed unpaired *t*-test with Welch’s correction, C1R-MICA transfectants versus monocytes).

We first analyzed MICA expression by total PBMC. *In vitro* stimulation with purified mono-nucleosomes leads to a slight MICA up-regulation by monocytes (**Figure 5A**), whereas no MICA expression was observed on lymphocytes. We next analyzed enriched monocytes instead of PBMC and we confirmed higher MICA expression by monocytes in response to mono-nucleosomes (**Figure 5B**). Similar results were obtained with purified monocytes (data not shown). Nevertheless, no sMICA was detected by ELISA in cell culture supernatants, even after stimulation (data not shown). Likewise, stimulation of enriched monocytes with GM-CSF slightly up-regulated MICA expression, whereas IL-17A (**Figure 5C**) or G-CSF (data not shown) had no effect. Similarly, a low MICA up-regulation was observed on PMN stimulated by G-CSF, but not by GM-CSF (**Figure 5D**), IL-17A or nucleosomes (data not shown). However, cytokine/nucleosome-stimulated monocytes and PMN did not release sMICA (data not shown). To explain why sMICA release is not observed, and whether high plasma membrane MICA expression may lead to sMICA release, we compared MICA expression and sMICA release by monocytes and C1R-MICA transfectants. Non-stimulated monocytes do express plasma membrane MICA after *in vitro* culture (as shown by the specific staining versus the isotype control, **Figure 6A**) and moderately up-regulate MICA in response to LPS, a potent MICA inducer (**Figure 6A**). In comparison, C1R-MICA transfectants express MICA at very high levels (**Figure 6B**). Moreover, while C1R-MICA transfectants released sMICA in cell culture supernatants, no sMICA was detected in monocyte culture supernatants, even after stimulation (**Figure 6C**), indicating that only extreme (either artificial or in some pathological environments) MICA expression, but not physiological MICA expression, may lead to detectable sMICA concentrations. Monocyte activation was confirmed by measuring IL-6 secretion (**Figure 6C**). Actually, no sMICA was detectable even at high cell concentration (up to 5.10^6^/ml) or at later time point (up to 48 hours, data not shown). We have also excluded that sMICA release is due to cell death. Indeed, treatment of PBMC, monocytes and PMN with staurosporine, actinomycin D, thapsigargin, H2O2 or a lysis buffer did not result in detectable sMICA in culture supernatants (data not shown).

### Extracellular Chromatin Mediates Natural Killer Cell Activation in a Monocyte-Dependent Manner

**Figure 7:**
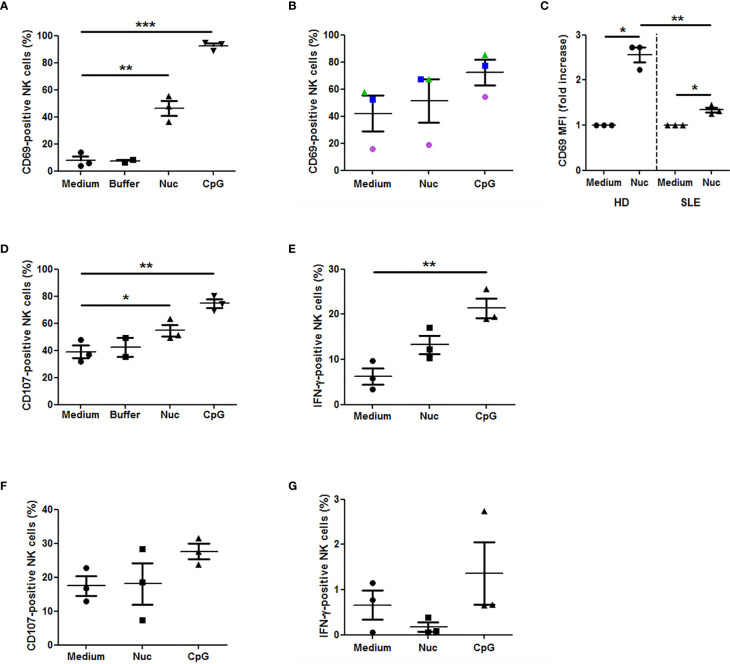
Extracellular chromatin triggers NK cell activation in PBMC. PBMC were isolated from healthy individuals (n = 3, **A, C–E**) or SLE patients (n = 3, **B, C, F, G**) and cultured in medium alone or supplemented with purified nucleosomes (Nuc, 25 µg/ml), its purification buffer or a TLR9 agonist (a synthetic oligonucleotide containing unmethylated CpG motifs, CpG). NK cell activation was analyzed by flow cytometry, gating on CD3^-^CD56^+^ cells as in **Supplementary Figure 2**. **(A–C)** CD69 expression by NK cells from healthy individuals **(A, C)** or SLE patients **(B, C)**. PBMC were cultured without K562 cells. The percentages of CD69-positive NK cells **(A, B)** or CD69 expression levels (C, mean fluorescence intensity (MFI) normalized to non-stimulated cells) are depicted. **(A)** **p < 0.01; ***p < 0.001 (two-tailed paired *t*-test). **(B)** Circles, triangles and squares represent PBMC of an independent donor analyzed in the three culture conditions. **(C)** *p < 0.05 (one sample *t*-test); **p < 0.01 (two-tailed unpaired *t*-test). **(D)** CD107 expression by NK cells from healthy individuals. PBMC were cultured in the presence of K562 target cells. *p < 0.05; **p < 0.01 (two-tailed paired *t*-test). **(E)** IFN-γ production by NK cells from healthy individuals. PBMC were cultured in the presence of K562 target cells. **p < 0.01 (two-tailed paired *t*-test). **(F)** CD107 expression by NK cells from SLE patients and analyzed as in **(D)**. **(G)** IFN-γ production by NK cells from SLE patients, analyzed as in **(E)**. Shown are pooled data from six independent experiments using PBMC from six independent donors. Each panel shows pooled data from either three independent healthy donors (HD) or three independent SLE patients, using five independent nucleosome preparations. Mean and SEM are depicted. The percentages of cells positive for the marker of interest or CD69 normalized levels among NK cells are shown.

**Figure 8:**
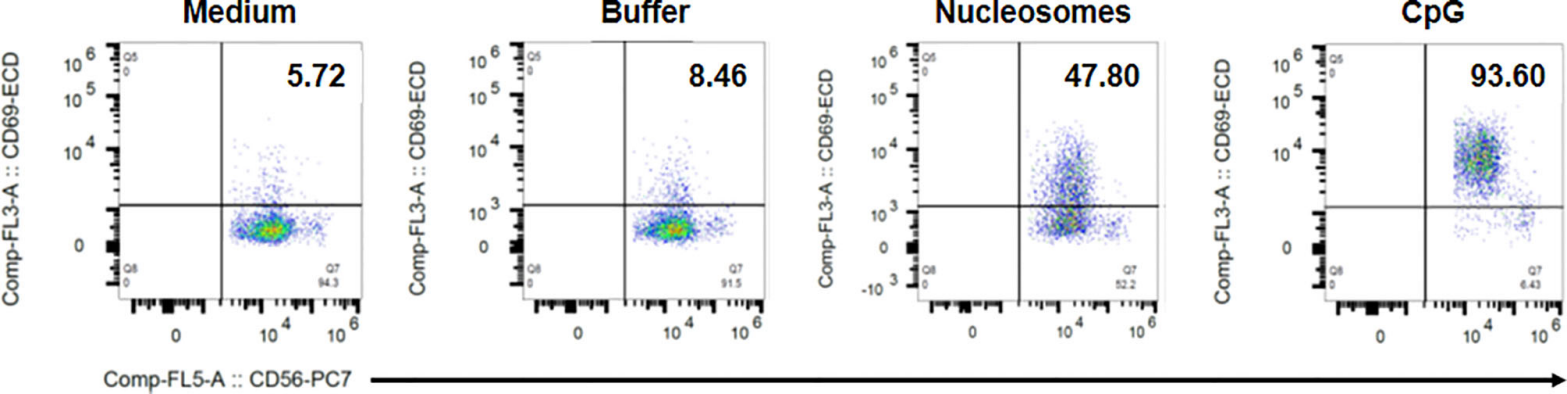
Representative flow cytometry dot plots showing nucleosome-induced CD69 up-regulation by NK cells in PBMC. PBMC were isolated from a healthy donor and cultured without K562 cells in medium alone, the nucleosome purification buffer, purified nucleosomes (25 µg/ml) or a TLR9 agonist (synthetic oligonucleotide containing unmethylated CpG motifs, CpG). Cells were gated on NK cells (CD3^-^CD56^+^ cells) as in **Supplementary Figure 2**. Dot plots depict CD56 staining (x axis) versus CD69 staining (y axis). The percentage of CD69-positive cells among NK cells is indicated for each condition in the upper right quadrant. Shown is one representative experiment of six independent experiments using different donors and five independent nucleosome preparations.

Because we observed that cell-free nucleosomes modulate MICA expression on monocytes, we next investigated whether nucleosome-stimulated monocytes influence NK cell activation and functions. We first analyzed NK cell phenotype in cultured PMBC stimulated with purified nucleosomes. The gating strategy is represented in **Supplementary Figure 2A**. In response to nucleosomes, NK cells from healthy individuals strongly up-regulated CD69 [**Figure 7A** (percentage of CD69-positive cells), p <0.01, two-tailed paired *t*-test and **Figure 7C** (CD69 expression levels), p <0.05, one sample *t*-test]. The nucleosome purification buffer had no effect. Representative flow cytometry dot plots are depicted in **Figure 8**. Monocytes are involved in nucleosome-induced CD69 up-regulation by NK cells, as depletion of monocytes from PBMC led to impaired CD69 up-regulation (**Supplementary Figure 3**). In contrast to nucleosomes, CD69 up-regulation by NK cells in response to a TLR9 agonist (e.g., CpG-ODN 2216) does not depend on the presence of monocytes. In agreement with a role of monocytes, stimulation of purified NK cells with nucleosomes did not lead to CD69 up-regulation or IFN-γ secretion (**Supplementary Figure 4**) or enhancement of cell activation, either when cultured in the absence or presence of IL-2, respectively (data not shown). To determine whether MICA expressed on nucleosome-activated monocytes is detected by NKG2D on NK cells, we measured NKG2D expression as NKG2D is down-regulated on NK cells after interacting with its ligands (34). We therefore determined the percentages of NKG2D-negative NK cells among PBMC (without K562 cells). Stimulation of PBMC with nucleosomes, as well as with the TLR9 agonist, led to an increased percentage of NKG2D-negative NK cells in the two individuals tested (**Supplementary Figure 5A**), suggesting that NKG2D is down-regulated after stimulation. Moreover, nucleosome-induced NKG2D down-regulation was abolished when PBMC were depleted of monocytes (**Supplementary Figure 5B**), suggesting that MICA expressed on monocytes is involved in NKG2D down-regulation. In contrast, NKG2D down-regulation in response to the TLR9 agonist was not abolished after monocyte depletion (**Supplementary Figure 5B**), which is in agreement with the observation that CD69 up-regulation by NK cells in response to the TLR9 agonist did not depend on the presence of monocytes, in contrast to nucleosomes (**Supplementary Figure 3**). CD69 expression by NK cells was also analyzed in PBMC isolated from SLE patients. Nucleosomes also slightly enhanced CD69 expression in all the three patients tested. Although NK cell activation was not statistically significant when analyzing the percentage of CD69-positive NK cells (**Figure 7B**), the level of CD69 expression was significantly increased in SLE NK cells stimulated with nucleosomes (**Figure 7C**, p <0.05, one sample *t*-test), indicating that SLE NK cells can still be partly activated. Actually, lupus NK cells present a strong basal activation level and seem to be exhausted, as shown by both the high CD69 expression in medium and the moderate response (not reaching significance) to the TLR9 agonist, respectively, suggesting that NK cells have been pre-activated *in vivo*. This probably explains the low response to nucleosomes. Indeed, although NK cells from both healthy individuals and SLE patients are activated by nucleosomes, cell activation was impaired in SLE patients as compared to healthy individuals (**Figure 7C**, p < 0.01, two-tailed unpaired *t*-test). NK cell functionality was then analyzed in the presence of K562 target cells. The gating strategy is represented in **Supplementary Figure 2B**. Nucleosomes, but not its purification buffer, stimulated NK cell degranulation (measured by CD107 expression by NK cells) in PBMC of healthy individuals (**Figure 7D**, p <0.05, two-tailed paired *t*-test), and CD107 up-regulation was dependent on the presence of monocytes (**Supplementary Figure 6**). As for CD69 up-regulation, CD107 up-regulation by NK cells in response to the TLR9 agonist (**Figure 7D**) was independent of the presence of monocytes, in contrast to nucleosomes (**Supplementary Figure 6**). Although not statistically significant, nucleosomes induced IFN-γ production by NK cells, as estimated by flow cytometry after intracellular cytokine staining, in PBMC from all the three healthy individuals tested (**Figure 7E**, p = 0.1). Finally, we measured NK cell functions in PBMC isolated from hydroxychloroquine-free SLE patients, in the presence of K562 target cells. No CD107 up-regulation was observed in response to nucleosomes, whereas the TLR9 agonist induced a low and not significant (p = 0.078) CD107 up-regulation (**Figure 7F**), suggesting exhaustion of NK cells from SLE patients. Likewise, no IFN-γ production by lupus NK cells was observed in response to nucleosomes and even in response to the TLR9 agonist (**Figure 7G**), especially when compared to IFN-γ production by NK cells from healthy individuals (**Figure 7E**), confirming exhaustion of NK cells from SLE patients probably as a result of long-term *in vivo* activation in this chronic disease.

## Discussion

Previous reports have suggested that NK cells are either protective or pathogenic in autoimmune diseases (in multiple sclerosis versus RA, respectively). Their role in SLE is still ambiguous. We have analyzed the NKG2D ligands MICA and MICB, because they have been shown to be expressed at sites of tissue inflammation, and because upon ligand binding, NKG2D stimulates effector functions.

In the present study, using several independent serum/plasma series (n = 204 independent donors), we show that circulating concentrations of both sMICA and sMICB are increased in SLE patients in comparison to healthy individuals. Elevated sMICA concentrations were previously reported in SLE, focusing on juvenile-onset SLE (26), but not compared to sMICB. Whether juvenile-onset SLE displays specific requirements for sMIC is unknown. Results were confirmed, although sMICA concentrations were described as low (27). Only one study reported recently elevated serum sMICA and sMICB concentrations in SLE patients in comparison to healthy individuals (35). Nevertheless, SLE patients were not compared to other autoimmune patients. In our study, SLE patients were compared to RA patients, as well as patients with leukemia. We also report for the first time that sMICB concentrations are increased in pSS patients, and we confirm data on sMICA as well. Indeed, higher concentrations of sMICA (only one study, and sMICB was not tested), were previously detected in the serum of pSS patients compared to healthy individuals (36). Interestingly, sMICA concentrations are associated with the development of anti-SSB and anti-RNP autoantibodies in our cohort of SLE patients. In agreement with our results, pSS is associated with high sMICA concentrations in patients with anti-SSA and/or anti-SSB antibodies compared to patients without anti-SSA and/or anti-SSB antibodies (36). Likewise, neonates exposed to anti-Ro/La antibodies have been recently shown to present higher frequencies of NK cells and especially IFN-γ-producing NK cells (37).

To understand the mechanisms leading to sMIC release in SLE, we also investigated potential triggers of MICA up-regulation and we observed that some cytokines increased MICA expression on both monocytes and PMN. Importantly, monocytes are known to differentiate in dendritic cells in SLE (38), whereas PMN are activated in patients (39). As shown previously, TLR4 stimulation induced MICA expression, whereas the lupus cytokine IFN-α had no effect (30). GM-CSF and G-CSF stimulated MICA expression on monocytes and PMN, respectively. GM-CSF concentrations, as well as the frequency of GM-CSF-secreting PBMC, are increased in SLE patients and the latter is correlated with anti-double-stranded DNA antibody titers (40, 41). Likewise, G-CSF administration to SLE patients induces flares (42). To mimic *in vivo* stimulation in patients, we have cultured monocytes from healthy individuals with nucleosomes and observed MICA induction, either on enriched monocytes or on monocytes in PBMC, leading to NK cell activation. Such a MIC induction with a lupus DAMP has not been reported so far. Monocytes from SLE patients may have a higher capacity to express MICA in response to nucleosomes. This hypothesis should be tested. Actually, a higher percentage of MIC-positive monocytes has been reported in SLE patients in comparison to healthy donors (43). Nevertheless, MICA up-regulation at the plasma membrane never led to sMICA release *in vitro*. Release of sMICA was only detectable with high expressing C1R-MICA transfectants. Reciprocally, C1R-mock transfectants, which express similar levels of MICA than monocytes, do not release detectable levels of sMICA (30). We also investigated whether cell death might lead to sMICA release by monocytes and PMN. Indeed, high apoptosis rates have been reported in SLE, especially in PMN, whereas necrosis can occur during tissue damage or secondary to impaired clearance of apoptotic cells. However, treating PBMC, monocytes or PMN with a variety of cell death-inducing agents did not induce sMICA release. Then, because both B lymphocytes and IFN-α are involved in SLE pathogenesis, we investigated whether they might be either the cellular source, or the triggering factor, of sMICA, respectively. Although we realize that the mechanisms regulating sMICA release might be different in SLE, we took advantage of two therapeutic approaches in other patient groups, namely Behcet patients with IFN-α therapy and RA patients with B-cell depletion therapy (although the size of the latter group was limited). Quantifying and comparing serum sMICA concentrations before and after therapy did not reveal modulation of sMICA. This analysis suggests at least that IFN-α (and maybe B lymphocytes) do not present an intrinsic capacity to influence sMICA release.

Finally, to better understand SLE physiopathology, we analyzed NK cells in PBMC after stimulation with nucleosomes. In healthy individuals, we show for the first time that nucleosomes activate NK cells and in a monocyte-dependent manner, indicating that nucleosome-mediated activation requires monocyte-NK cell interplay. Indeed, NK cell activation was impaired in monocyte-depleted PBMC and purified NK cells (devoid of monocytes) do not respond to nucleosomes. However, NK cell activation in response to a TLR9 agonist was not dependent on monocytes. Nucleosome-induced NK cell activation manifested as CD69 up-regulation and, in the presence of K562 target cells, as enhanced degranulation and a tendency to produce more IFN-γ. Interestingly, NK cells with higher expression of CD69 and an increased capacity to produce IFN-γ have been reported in active SLE (17). Our results suggest that the monocyte-NK cell interplay occurs, at least partly, *via* MICA-NKG2D interaction, as we observed an NKG2D down-regulation on NK cells. We have previously reported that this mechanism is involved in NK cell activation following activation of monocytes by LPS (30). Moreover, NK cell activation was only observed in the presence of both monocytes and nucleosomes, and nucleosomes enhance MICA expression on monocytes. Although MICA expression in response to nucleosomes is mild, such expression was previously shown to efficiently induce NK cell activation in a NKG2D-dependent manner (30). Cell activation might also be partially helped by other interactions, as e.g. AICL-NKp80 interaction which stimulates monocyte-NK cell crosstalk (44). Using PBMC of SLE patients, NK cell activation was also observed, either with nucleosomes or in response to a TLR9 agonist, but was however impaired as compared to healthy individuals. Those results indicate that lupus NK cells are exhausted and suggest that they have been continuously stimulated *in vivo*, e.g. by nucleosomes. Indeed, circulating nucleosomes are usually detectable in SLE patients but not in healthy individuals. Nevertheless, SLE NK cells might also respond less because SLE PBMC release higher amounts of soluble MIC, which would act as a decoy receptor. We did not detect sMICA in cell culture supernatants, however sera with low sMICA concentration (undetectable by ELISA) have been shown to down-regulate NKG2D (21).

MICA may be involved at two levels in SLE, either as a plasma membrane or a soluble molecule. Moreover, sMICA may act *via* different mechanisms on NK and T cells. MICA expression is increased in the kidneys of SLE patients, and in lupus mice NKG2D ligand expression in the kidney correlates with infiltrating NK cells displaying high IFN-γ production (45). However, although concentrations of circulating sMICA were not higher in SLE patients in comparison to healthy individuals, sMICA concentrations were shown to be increased in SLE patients with active nephritis versus patients without nephritis and an inverse trend, although not significant, with blood NK cell concentrations was observed (46). Those two studies suggest a pathogenic role for MICA or sMICA, associated with increased tissue NK cells or decreased blood NK cells. One could also argue that activated NK cells migrate to inflamed tissue in active SLE, explaining the drop a circulating NK cells. In addition, MICA and sMICA act not only on NK cells, but also on T cells. Thus, persistent MICA expression drives the proliferative expansion of NKG2D^+^CD4^+^ T lymphocytes having negative regulatory functions (47). On the opposite, in RA, MIC-expressing synoviocytes expand autoreactive NKG2D-expressing CD4^+^CD28^-^ T cells (25). Similarly, opposite activities have been reported for sMICA/B. Soluble MICA has been shown to down-modulate NKG2D on CD8^+^ T cells (and presumably also on NK cells, although it was not tested) in breast cancer, working here as a decoy molecule (21). In RA, serum sMICA is unable to down-regulate NKG2D due to the presence of TNF and IL-15 (25). More recently, it was suggested that sMICB drives expansion of a T-cell population in juvenile SLE, however with suppressive functions (27). Thus, sMICA/B inhibit or stimulate T lymphocytes. Whether sMICA/B are able to trigger effector functions of NK cells is unknown.

CD4^+^NKG2D^+^ T cells have also been reported in SLE (43). Moreover, this study reported that lupus monocytes aberrantly express MIC, without distinguishing MICA from MICB, and that sera from SLE patients induce MIC expression by monocytes from healthy individuals in a partly IFN-γ-dependent manner (suggesting that other circulating factors are involved, e.g. nucleosomes). Interaction between MIC^+^ monocytes and NKG2D^+^CD4^+^ T cells leads to T-cell activation and IFN-γ production in a MIC-dependent manner. However, NK cells were not analyzed. It should be noted that we did not observe MICA up-regulation by normal monocytes cultured with sera of SLE patients (data not shown). On the contrary, in juvenile-onset SLE, monocytes express lower levels of MICA/B than monocytes from healthy controls (27). Thus, contradictory data were also reported for MICA expression by monocytes in SLE. Moreover, no correlation was observed in a larger cohort between CD4^+^CD28^-^NKG2D^+^ T cells and autoimmunity (48).

Contrasting data concern also gene association studies. *MICA* polymorphisms have been either negatively or positively associated with SLE in the Japanese population, and a recombinant MICA protein variant associated with SLE decreases NKG2D expression and cytotoxicity by NK cells *in vitro*, but enhances IFN-γ production (49). Positive and negative associations of *MICA* polymorphisms with SLE were also reported in the Italian population (50), whereas no primary association was observed between *MICA* polymorphisms and SLE in the Spanish population (51). No association was observed in a meta-analysis between *MICA* polymorphisms and SLE susceptibility, regardless of the ethnic group (52), although not all the *MICA* polymorphisms previously reported were investigated.

In conclusion, we report high concentrations of soluble MICA/B in the circulation of SLE patients, plasma membrane MICA expression by monocytes and PMN activated by nucleosomes or cytokines, and NK cell activation by nucleosome-stimulated monocytes *via* NKG2D. A regulation of plasma membrane MICA expression and a balance between plasma membrane MICA and MICA shedding is probably key to control SLE development, *via* the modulation of NK cell activity and the expansion of T cells, some of which being suppressive. An excess of proteases, especially metalloproteases such as ADAM, might be involved in sMICA/B release in SLE, as reported with tumor cells (24, 53). However, sMICA concentrations detected might be too low to block cell activation. Whether it is the consequence of plasma membrane or soluble MICA, we observed a down-regulation of NKG2D after stimulation with nucleosomes, which was associated with NK cell activation. Finally, NKG2D has also been linked to tissue inflammation and to the promotion of autoimmunity, in celiac disease, by arming cytotoxic T lymphocytes. Upon recognition of MIC^+^ target cells, cytotoxic T lymphocytes release arachidonic acid (54).

## Data Availability Statement

The raw data supporting the conclusions of this article will be made available by the authors, without undue reservation.

## Ethics Statement

The studies involving human participants were reviewed and approved by CPP Paris Ile de France (NI-2016-11-01, Bobigny), CPP IDF VI (June 26th, 2012, Paris), 146/2001V, 386/2006V, and 647/2016B02 (Tübingen), Etablissement Français du Sang (EFS, 18/EFS/033). The patients/participants provided their written informed consent to participate in this study.

## Author Contributions

All authors were involved in drafting the manuscript. PD designed the research, performed most of the experiments, coordinated the study, analyzed and interpreted data and wrote the manuscript. BH performed part of the experiments, analyzed and interpreted data. MR, NT, JM, and MB performed part of the experiments and analyzed data. VV and AS analyzed the data. ZA selected patients and analyzed the data. RK and IK selected patients, analyzed and interpreted data. All authors contributed to the article and approved the submitted version.

## Funding

This work was supported by INSERM and the University Sorbonne Paris Nord (formerly University of Paris 13) as well as by grants from the University of Paris 13 (“Bonus Qualité Recherche”, BQR), from the German Research Foundation [Deutsche Forschungsgemeinschaft (DFG), DE 879/1-2], from the University of Tübingen (Interdisziplinäres Zentrum für klinische Forschung Tübingen (IZKF-Nachwuchsgruppe), 1604-0-0 and 1604-0-1; and *f*ortüne (Forschungsprogramm der Tübinger Medizinischen Fakultät), 1451-0-0) and from the Fritz-Thyssen Foundation (Az. 10.10.2.128) to PD. MR was partly supported by a fellowship from Fondation Arthritis.

## Conflict of Interest

The authors declare that the research was conducted in the absence of any commercial or financial relationships that could be construed as a potential conflict of interest.
