# Supplementary material for: Increased Concentrations of Circulating Soluble MHC Class I-Related Chain A (sMICA) and sMICB and Modulation of Plasma Membrane MICA Expression: Potential Mechanisms and Correlation With Natural Killer Cell Activity in Systemic Lupus Erythematosus
Source: Front Immunol. 2021 May 3;12:633658. doi: 10.3389/fimmu.2021.633658 (PMC8126610; doi:10.3389/fimmu.2021.633658)
Supplement: Supplementary file 1 [file DataSheet_1.pdf]

Supplementary Material

Supplementary Figures

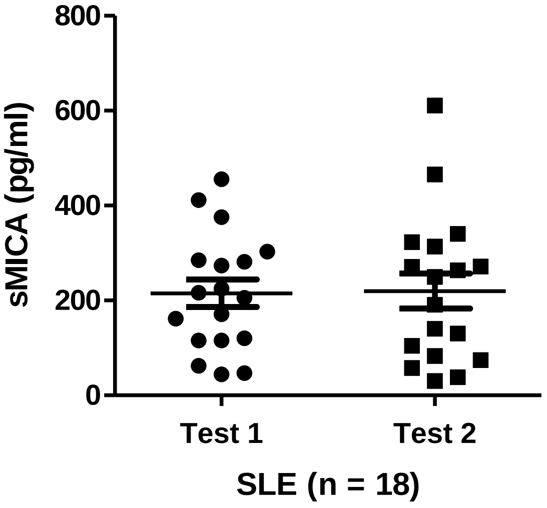

Supplementary Figure 1. Reproducibility of sMICA quantification. Sera from 18 SLE patients presented in Figure 1 were tested by ELISA at two different time points to measure sMICA concentrations. Each dot represents an independent patient. Mean and SEM of all donors are shown.

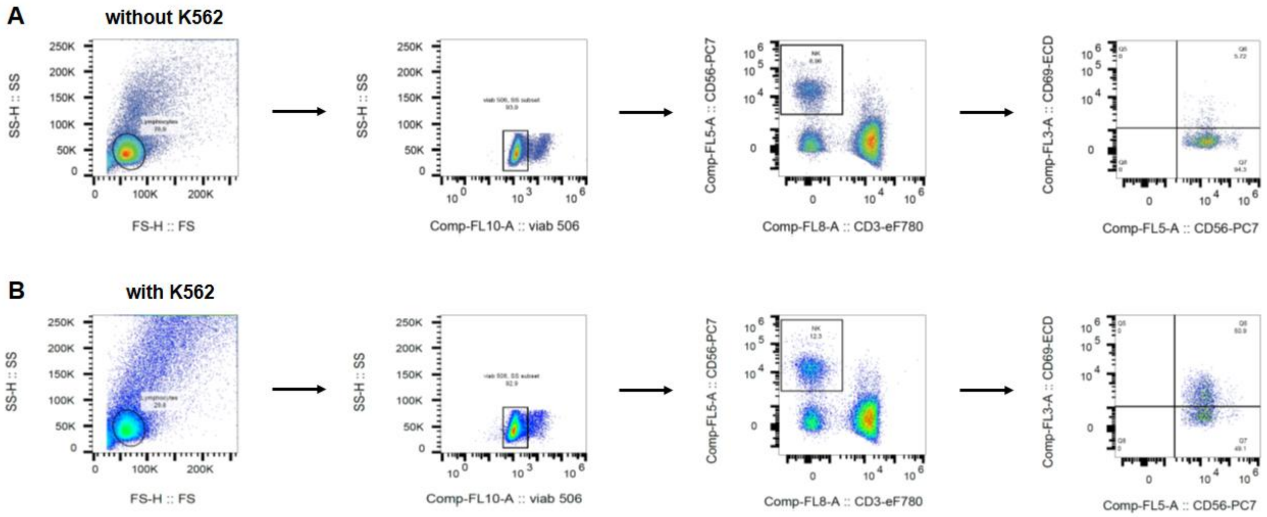

Supplementary Figure 2. Flow cytometry gating strategy to analyze NK cells in PBMC. Isolated PBMC were cultured alone (A) or with K562 target cells (B) and analyzed by flow cytometry. In each panel, cells were first gated (left) according to size (FS-H) and granularity (SS-H) and successively gated (from left to right) on living cells (viab), then on $CD3^{-}CD56^{+}$ cells (i.e., NK cells) and then analyzed for the marker of interest versus CD56. CD69 staining is show as an example, but the same approach was used for the other analyzed markers. Shown is one representative experiment of seven experiments using independent donors.

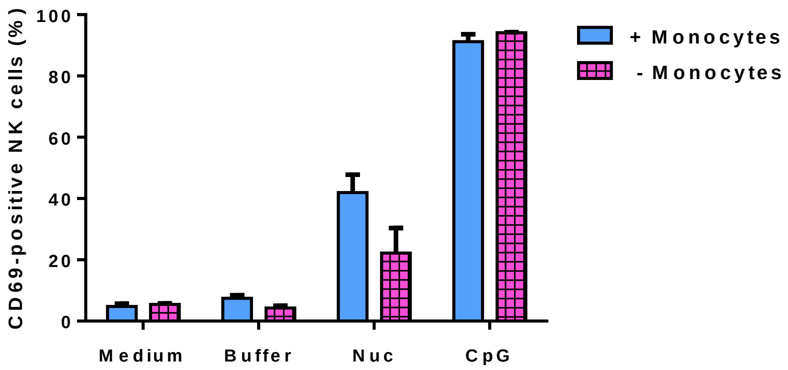

Supplementary Figure 3. Monocytes support nucleosome-induced CD69 up-regulation by NK cells in PBMC. PBMC were freshly isolated from two healthy donors and depleted (hatched pink bars) or not (blue bars) of monocytes by magnetic sorting using CD14-positive beads. Cells were then cultured without K562 cells in medium alone, the nucleosome purification buffer, purified nucleosomes (Nuc, 25 µg/ml) or a TLR9 agonist (CpG). CD69 expression was analyzed by flow cytometry on NK cells ($CD3^{-}CD56^{+}$ cells) gated as in Supplementary Figure 2. Shown are pooled data from two independent experiments using PBMC from two independent healthy donors. Mean and SEM are depicted.

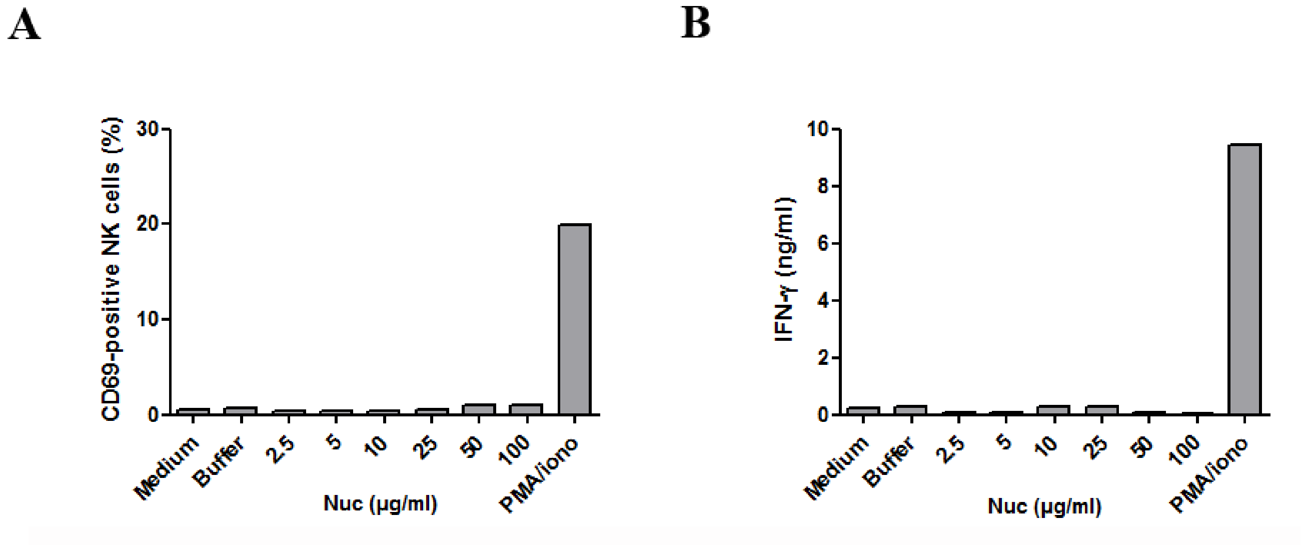

Supplementary Figure 4. Purified NK cells do not up-regulate CD69 or secrete IFN-γ in response to nucleosomes. PBMC were isolated from a healthy individual and NK cells were purified by negative selection using magnetic beads. NK cells were then cultured (without K562 cells) in medium alone, the nucleosome purification buffer, purified nucleosomes (Nuc) or PMA/ionomycine (iono) as a positive control. **(A)** CD69 expression was analyzed by flow cytometry. **(B)** IFN-γ secretion was estimated by ELISA. One representative experiment of five independent experiments using PBMC from five independent donors and independent nucleosome preparations is shown.

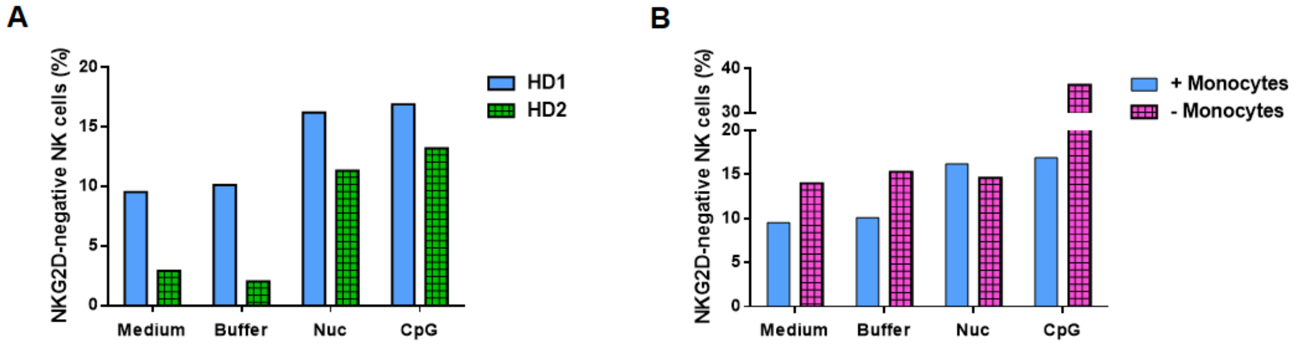

Supplementary Figure 5. Nucleosome-activated monocytes trigger NKG2D down-regulation by NK cells in PBMC. (A) PBMC were freshly isolated from healthy donors (HD) and cultured without K562 cells in medium alone, the nucleosome purification buffer, purified nucleosomes (Nuc, 25 µg/ml) or a TLR9 agonist (CpG), and then analyzed by flow cytometry. The percentage of NKG2D-negative NK cells among total NK cells ($CD3^{-}CD56^{+}$ cells gated as in Supplementary Figure 2) is depicted. Two independent experiments with PBMC from two independent healthy donors are shown. **(B)** PBMC were isolated from one healthy donor and depleted (hatched pink bars) or not (blue bars) of monocytes by magnetic sorting using CD14-positive beads. Cells were then cultured without K562 cells in medium alone, the nucleosome purification buffer, purified nucleosomes (Nuc, 25 µg/ml) or a TLR9 agonist (CpG). The percentage of NKG2D-negative NK cells among total NK cells was estimated as in (A). Results are from one experiment.

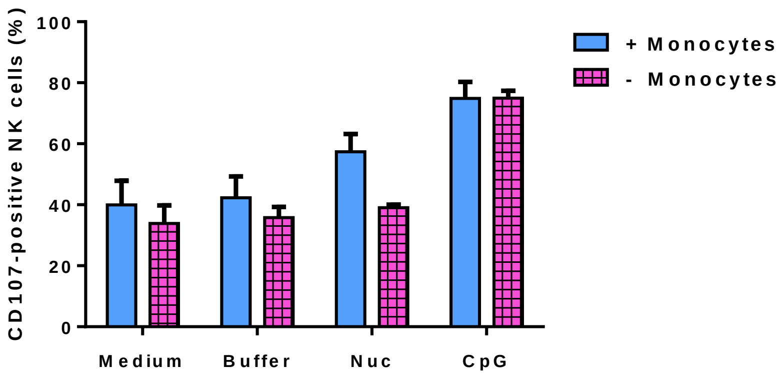

Supplementary Figure 6. Monocytes support nucleosome-induced CD107 up-regulation by NK cells in PBMC. PBMC were freshly isolated from two healthy donors and depleted (hatched pink bars) or not (blue bars) of monocytes by magnetic sorting using CD14-positive beads. Cells were then cultured in the presence of K562 target cells in medium alone, the nucleosome purification buffer, purified nucleosomes (Nuc, 25 µg/ml) or a TLR9 agonist (CpG). CD107 expression was analyzed by flow cytometry on NK cells ($CD3^{-}CD56^{+}$ cells) gated as in Supplementary Figure 2. Shown are pooled data from two independent experiments using PBMC from two independent healthy donors and two independent nucleosome preparations. Mean and SEM are depicted.
